# Isolation and characterization of *Escherichia coli* serotype O157:H7 and other verotoxin-producing *E. coli* in healthy Indian cattle

**DOI:** 10.14202/vetworld.2020.2269-2274

**Published:** 2020-10-29

**Authors:** Dasharath B. Shinde, Surbhi Singhvi, Santosh S. Koratkar, Sunil D. Saroj

**Affiliations:** Symbiosis School of Biological Sciences, Symbiosis International (Deemed University), Pune, Maharashtra, India

**Keywords:** cattle, *Escherichia coli* O157:H7, shiga toxin, vero cells, verotoxin

## Abstract

**Background and Aim:**

Cattle are the main reservoir of *Escherichia coli* O157:H7 and other verotoxigenic *E. coli* (VTEC); therefore, there is an increased risk of infection to humans by either direct or indirect mode of transmissions. However, the prevalence of *E. coli* O157:H7 in the healthy cattle population of India is yet to be ascertained. This study aimed to screen the dairy cattle in and around Pune, Maharashtra, India, for verotoxin-producing *E. coli* O157:H7.

**Materials and Methods:**

A total of 257 rectal swabs were collected from 15 different organized and unorganized dairy farms of Pune during the period, January-March 2015. The screening involved enrichment in EC broth followed by differential identification on MacConkey sorbitol agar. The presumptive positive isolates were further confirmed by multiplex polymerase chain reaction (PCR) using primers specific to *rfbE* (O157), *fliC* (H7), *VT*1 (MK1), and *VT*2 (MK2). Vero-toxicity and antibiotic sensitivity were examined in PCR confirmed isolates.

**Results:**

Out of the 257 samples analyzed, 1.9% (2/105) were positive for O157:H7 and 39% (41/105) were positive for VTEC. Two PCR confirmed positive O157:H7 strains and two randomly selected PCR-positive VT strains exhibited *in vitro* cytopathic effect on Vero cells on day-7 post-inoculation. Antibiotic sensitivity profiling of O157:H7 strains exhibited resistance against penicillin G, kanamycin, ampicillin, tetracycline, gentamycin, cefotaxime, streptomycin, and piperacillin.

**Conclusion:**

These findings reveal the presence of pathogenic *E. coli* O157:H7 in the healthy cattle of Pune; in a situation, wherein regular surveillance for O157:H7 is not a norm. Therefore, the findings presented herein warrant routine surveillance and public awareness to prevent the transfer of such pathogens and manage health risks to the public.

## Introduction

Verotoxin-producing *Escherichia coli*, also known as Shiga toxin-producing *E. coli*, is an important foodborne and zoonotic pathogen. It is associated with several human diseases which include hemorrhagic colitis, diarrhea, thrombotic thrombocytopenic purpura (TPP), and potentially fatal hemolytic uremic syndrome (HUS) [1,2]. The most predominant serotype of verotoxigenic *E. coli* (VTEC) is *E. coli* O157 infecting more than 73,000 people, causative of 61 deaths each year, and an average, 500 outbreaks in the United States alone [3,4]. In the United States from 2003 to 2012, *E. coli* O157 was causative for 390 outbreaks, 4928 illnesses, 1272 hospitalizations, and 33 deaths [5]. In Canada, a systematic investigation of the outbreaks concluded that *E. coli* O157:H7 infections are common and linked to mechanically tenderized beef. The findings prompted amendments to the Canada Food and Drug Regulations about the requirement for mechanically tenderized beef to be labeled and provide safe cooking instructions to consumers [6].

Transmission of *E. coli* O157 takes place through the fecal–oral route in which contact with the animal and visit to the farm has a predominant role [7]. Cattle are considered as the principal reservoir of VTEC O157:H7; therefore, it functions as a potential source for transmission of VTEC O157:H7 to humans by direct or indirect route [8]. VTEC has been isolated from healthy intestines of sheep, deer, and buffalo; the distribution is not restricted to a particular geographical area, and among the isolates, serotype O157:H7 is mostly associated with severe human infections [9]. The virulence attributed to O157:H7 is due to the secretion of Shiga toxins (Stx), which inhibits protein synthesis of the host cells, ultimately leading to cell death. The Shiga toxin subtypes, Stx1, and Stx2, are involved in the signal transduction and secretion pathway [7,10].

A limited number of investigations to determine the prevalence of O157:H7 in India have been conducted. In 2002, Khan *et al*. [11] investigated STEC in hospitalized diarrhea patients, healthy cattle, and beef samples from Calcutta. In 2006, Manna *et al*. [12] reported the occurrence of verotoxin-producing, antibiotic-resistant *E. coli* O157 in cattle population from West Bengal. Hazarika *et al*. [13] reported the prevalence of VTEC O157:H7 in beef from Bareilly.

Regular surveillance for O157:H7 is not a practice in India. Additional information on the prevalence of O157:H7 in a healthy cattle population will assist public health authorities in device policies to curtail its spread and prevent related infections. The study aimed to understand the prevalence of verotoxin-producing *E. coli* O157:H7 from healthy cattle in Pune.

## Materials and Methods

### Ethical approval and informed consent

Ethical approval for such type of study is not required. However, cattle were handled carefully during rectal swab collection. Prior consent was taken from owner of the cattle farm. The privacy and confidentiality of personal information of participating farms and farm owners are not disclosed in the manuscript.

### Reagents and chemicals

Chemicals, bacterial and cell culture media, antibiotics, and FBS used in the present study were procured from HiMedia Laboratories Pvt. Ltd., India. Polymerase chain reaction (PCR) primers were synthesized from BioResource Biotech Pvt. Ltd., India. Other PCR reagents such as dNTPs, Taq DNA polymerase, and PCR buffers were procured from Bangalore Genei, Bengaluru, India. The chemicals used were of the highest purity available.

### Sample collection

Rectal swabs of cattle were collected from various organized and unorganized farms in and around Pune district during the period January-March 2015. Rectal swabs were collected from 15 different dairy cattle (cow/buffalo) using the method previously described by Bessalah *et al*. [14]. Drinking water samples from six different farms were also collected and tested for the presence of VTEC. Details of the sample collection are provided in Table-1.

**Table 1:** Details of rectal swabs collection from different farms in and around Pune, India.

Farm	Male		Female		Total
	
	Adult	Calf	Adult	Calf	
1	-	-	-	20	20
2	-	-	1	21	22
3	-	-	5	3	8
4	-	1	11	3	15
5	1 (B)	2	8	10	21
6	1	8	3	8	20
7	1 (B)	4 (B)	8 (B)	7 (B)	20
8	-	2	8	10	21*
9	-	4	-	15	19
10	1	-	8	1	11*
11	1	-	27, 3 (B)	5, 1 (B)	38*
12	-	-	7, 1(B)	3	12*
13	-	-	8	1	10*
14	-	-	8	2	10
15	-	-	6	3	10*

* Represents one additional drinking water sample collected from farm and B in brackets, that is, (B) represents the respective rectal swab collected from buffalo and rest of all are cow swabs

### Enrichment and screening

Rectal swabs were directly collected into 3 ml EC Medium (HiMedia-M127) and kept on ice while transferring to the laboratory [12]. Samples were incubated overnight at 37°C without shaking and streaked on MacConkey sorbitol agar (HiMedia-M298) and incubated at 37°C overnight. *E. coli* isolates were differentiated based on the ability to ferment sorbitol. Five representative colonies were picked from each plate and further purified by three passages on SMAC agar. The isolates were subjected to Gram staining and IMViC (Indole, Methyl Red, Voges-Proskauer test, and Citrate utilization test) [15]. The presumptive positive *E. coli* were further confirmed by PCR.

### DNA extraction

DNA was extracted using the boiling method as described by Bessalah *et al*.[14] and Blanco *et al* [16] with few modifications. Briefly, colonies from the SMAC plate were suspended in 1 mL sterile deionized water and placed in a water bath at 100^o^C for 10 min. The supernatant was used as template DNA.

### Oligonucleotide primers used in the study

Primers specific to O157, H7, and *VT* genes were used. The details are listed in Table-1 [17,18].

### PCR

PCR was performed as described earlier by Nagano *et al.*, [19]. Briefly, a total of 20 μL reaction mixture containing 1L of template DNA, 5 μM each primer, 2.5 mM dNTPs, and 1 U Taq DNA polymerase were used. The thermal cycling conditions are shown in Table-2 [18]. The amplified product was analyzed by electrophoresis on 1.5% agarose gel, stained with ethidium bromide (EtBr), and visualized under UV transillumination.

**Table 2:** The PCR primers and thermal cycling conditions used for the amplification of verotoxin-producing genes in *Escherichia coli* [18].

Primers	Target gene	Amplicon (bp)	PCR conditions		

			Denaturing	Annealing	Extension
H7-F	*fliC* gene of H7	560	90°C 2 min	60°C 1 min	72°C 90 s*
H7-R					
O157-F	*rfbE* gene of O157	678	90°C 2 min	60°C 1 min	72°C 90 s*
O157-R					
MK-1	*VT* gene	227	90°C 2 min	60°C 1 min	72°C 90 s*
MK-2					

* Represents final extension of 10 min at 72°C after 35 PCR cycles. PCR=Polymerase chain reaction

### Vero cell assay

Vero cells were procured from National Center for Cell Sciences, Pune, grown and maintained in Minimal Essential Medium (MEM, AL047S) supplemented with 10% fetal bovine serum (FBS, RM9955) and 1% antibiotic (penicillin and streptomycin, A001) [20].

### Preparation of cell-free culture filtrates

PCR-positive O157:H7 and other VTEC isolates were inoculated in nutrient broth and incubated at 37°C overnight with constant shaking. The supernatant obtained after centrifugation at 13,000 RPM for 15 min was filtered using 0.22 μm syringe filters. The bacterial pellet obtained was resuspended in 1 mL PBS and sonicated to obtain cell lysate. The lysate was centrifuged to remove debris at 13,000 rpm for 10 min at 4°C. The cell lysate was filter sterilized using 0.22 μm filters. Both the culture supernatants and cell lysates were used for the cytotoxicity assay [11,21].

### Cytotoxicity assay

The cytotoxic effect of VTEC was examined on Vero cells in a 96-well plate. The cell-free culture filtrates (20 μL of supernatant/100 μL of cell lysate) were incubated with Vero cells in a 100 μL MEM. The Vero cells were microscopically observed for 7 days, and cytopathic effects (CPE) were recorded [22,23].

### Antimicrobial susceptibility testing

PCR-positive O157 and O157:H7 isolates were characterized for antibiotic susceptibility on Mueller-Hinton agar (MH agar) plates by the disk diffusion method using 14 different antibiotic disks (penicillin G, kanamycin, piperacillin, amikacin, gentamycin, cefotaxime, streptomycin, chloramphenicol, tetracycline, cotrimoxazole, azithromycin, nalidixic acid, ampicillin, and nitrofurantoin) [24]. The results of disk diffusion were interpreted using the standard guidelines of the Clinical and Laboratory Standards Institute.

## Results

A total of 257 rectal swabs of healthy dairy cattle (cow/buffalo) were collected from 15 different organized/unorganized farms in and around Pune, Maharashtra, during the period January-March 2015 (Table-1).

### Screening of O157:H7 and VTEC

The initial screening of rectal swabs for *E. coli* O157:H7 was performed on MacConkey Sorbitol Agar (SMAC). Out of 257 samples, 105 were positive for sorbitol non-fermenting *E. coli*. The sorbitol non-fermenting colonies were further subjected to biochemical tests. The presumptive positive isolates were further confirmed for O157, H7, and *VT* genes using multiplex PCR. All the sorbitol non-fermenting isolates were positive for the indole test and methyl red test and negative for Voges-Proskauer. Isolates were further tested for growth characteristics on eosin methylene blue (EMB) agar. On EMB agar the colonies appeared dark centered with a green metallic sheen. *E.coli* produces dark centered colonies on EMB agar with metallic sheen due to lactose fermentation and acid production. All 105 isolates screened on SMAC were screened in a multiplex PCR for O157, H7, and *VT* genes using specific primers, as mentioned in Table-2. The multiplex PCR using three sets of primers showed three distinct bands. Out of 257 samples tested, H7 was detected in 29/105 (27.61%), O157 was detected in 2/105 (1.9%), and *VT* was detected in 41/105 (39.04%) samples. PCR confirmed *E. coli* O157:H7 was detected in 1/108 adult cow and 1/14 adult buffalo; whereas, none of the samples from cow and buffalo calves were tested positive. PCR confirmed *E. coli* H7 was detected in 22/225 cow and 7/26 buffalo. PCR confirmed *E. coli* *VT* was detected in 34/225 cow and 7/26 buffalo (Table-3).

**Table 3:** Polymerase chain reaction screening for three different verotoxin-producing genes (O157, H7, and VT) in *Escherichia coli* isolates from cattle and buffalo.

Gene names	Cattle				Buffalo				Drinking water samples (6)
	
	Calf		Adult		Calf		Adult		
			
	Male (17)	Female (100)	Male (3)	Female (105)	Male (4)	Female (8)	Male (2)	Female (12)	
O157	0	0	1	0	0	0	1	0	0
H7	4	6	0	12	0	2	0	5	0
VT	4	12	2	16	0	2	1	4	0

### Vero cell assay

The *VT* and O157:H7-positive isolates were further examined for cytotoxicity using Vero cells. All samples showed a cytotoxic effect on Vero cells, which may be due to the presence of verotoxins. CPE was observed in Vero cells exposed to both the VTEC and O157:H7 isolates on post-inoculation day-7.

### Antimicrobial susceptibility testing

The antimicrobial susceptibility of isolates was examined by the disk diffusion method using 14 different antibiotics, as described in the methods section. It was observed that the O157-positive isolate was resistant to penicillin G, piperacillin, tetracycline, cotrimoxazole, ampicillin, and nitrofurantoin; whereas, O157:H7 isolates exhibited resistance to penicillin G, piperacillin, ampicillin, and nitrofurantoin (Table-4).

**Table 4:** Antibiotic susceptibility of *E. coli* O157 and O157:H7 isolates.

S. No.	Antibiotics (μg/disk)	Representative *E. coli* isolates	

		O157	O157:H7
1.	Penicillin G (30)	R	R
2.	Kanamycin (30)	S	S
3.	Piperacillin (100)	R	R
4.	Amikacin (30)	S	S
5.	Gentamycin (10)	S	S
6.	Cefotaxime (30)	I	I
7.	Streptomycin (10)	S	S
8.	Chloramphenicol (30)	S	S
9.	Tetracycline (30)	R	S
10.	Cotrimoxazole (25)	R	S
11.	Azithromycin (15)	S	S
12.	Nalidixic acid (30)	S	S
13.	Ampicillin (10)	R	R
14.	Nitrofurantoin (300)	R	R

S=Sensitive, I=Intermediate, and R=Resistant as per the standard guidelines of the Clinical and Laboratory Standards Institute. *E. coli=Escherichia coli*

## Discussion

VTEC has evolved from a clinically restricted strain to a global public health concern during the past few decades and has been prevalent in food products of more than 30 countries. VTEC is the causative of a spectrum of illness in humans, from asymptomatic infections to severe bloody diarrhea and, in some instances, may lead to life-threatening conditions. The information on the prevalence of VTEC strain in India is rare. However, O157:H7 is the most isolated strain among VTEC-related infections. In addition to O157:H7, several other serotypes of VTEC have been isolated from humans, food, and animal sources; however, they are not characterized in detail for their virulence attributes. *E. coli* O157:H7 is the most prevalent VTEC in foodborne outbreaks in North America, Europe, and Japan [25].

Cattle are the main reservoir of *E. coli* O157 and non-O157 VTEC; therefore, frequently observed as a zoonotic agent in human infection. Domestic cattle farming is a widespread household practice in rural India; due to which individuals can establish close contact with the cattle during daily routines. Therefore, sizeable Indian population is subjected to a risk of contracting *E. coli* O157:H7. On the contrary, in India, VTEC infections are not a significant problem; maybe, due to the presence of acquired antibodies at an early age and the cooking practices followed. In India, the majority of the population do not consume beef for religious reasons; therefore, the possible mode of VTEC transmission is through direct and indirect contact of cattle waste with vegetables, fruits, and drinking water. *E. coli* O157:H7 has been isolated from a variety of food products marketed in India.

To understand the prevalence of *E. coli* O157:H7, rectal swabs were collected from 15 different organized and unorganized farms and streaked onto the SMAC; the non-sorbitol fermenting colonies were selected. During the initial screening, 40.85% of samples from the cattle and water were presumptive positive. The representative colonies from each presumptive positive sample were further confirmed by multiplex PCR [19]. O157, H7, and *VT* were confirmed in 2/105 (1.90%), 29/105 (27.61%), and 41/105 (39.04%) of samples, respectively. The data reveal the presence of O157 and VTEC strains in the healthy cattle from Pune. The frequent isolation of *E. coli* O157:H7 from healthy cattle is a serious public health concern. A similar study to understand the prevalence of O157:H7 in cattle by Leung *et al.*, [26] in Hong Kong reported 409/986 (41.5%) and 9/409 (2.20%) samples positive for VTEC and O157:H7. Similar observations were made by Leung *et al.*, [26]; wherein, O157:H7 was detected in 17/1226 (1.34%) of the samples analyzed.

In the present study, the PCR confirmed VTEC exhibited cytotoxicity on Vero cells. The occurrence of VTEC in healthy cattle in Pune is of serious health concern. In a previous report, the presence of cytotoxic VTEC in the healthy population of cattle from West Bengal has been reported by Khan *et al.*, [21].

All *E. coli* isolates obtained were resistant to at least one or more antimicrobial agents used in this study. Resistance toward penicillin G, piperacillin, tetracycline, cotrimoxazole, ampicillin, and nitrofurantoin was observed. A similar pattern of antimicrobial resistance in *E. coli* isolates from cattle has been reported in other studies [12,27,28]. High occurrence of antimicrobial resistance in *E. coli* O157 isolates from cattle may confer a selective advantage toward intestinal colonization. Furthermore, this further leads to increased fecal shedding of antimicrobial-resistant *E. coli* O157:H7. Although a recent study on meta-analysis showed no association between the use of antibiotics and the incidence of HUS, the use of antibiotics for the treatment is unclear and more strain-specific trails are recommended [29].

## Conclusion

Our findings suggest that dairy cattle are a frequent reservoir of *E. coli* serotype O157:H7 and another Shiga toxin *E. coli*. The VTEC isolates were resistant to the most common antimicrobials and harbor genes for cytotoxicity. The findings warrant additional screening studies of VTEC in both the organized and unorganized cattle farming sector in India to understand the prevalence and characterization of virulence attributes.

## Authors’ Contributions

SSK designed the experiments and supervised the study. DBS and SS collected the samples, performed experiments, and analyzed the results. DBS and SSK prepared the draft manuscript. SDS helped in critical review and data representation of the manuscript. All authors approved this manuscript for publication. All authors read and approved the final manuscript.
